# Appealing to the Republic of Letters: An Autopsy of Anti-venereal Trials in Eighteenth-century Mexico

**DOI:** 10.1093/shm/hkt045

**Published:** 2013-10-25

**Authors:** Fiona Clark

**Keywords:** venereal disease, Mexico, republic of letters, eighteenth century, France

## Abstract

This study analyses the narrative elements of a little-known report into anti-venereal trials written by an Irish military physician-surgeon, Daniel O'Sullivan (1760–*c*.1797). It explores the way in which O'Sullivan as the narrator of the *Historico-critical report* creates medical heroes and anti-heroes as a means to criticise procedures initiated by staff in the Hospital General de San Andrés, Mexico City. The resulting work depicts a much less positive picture of medical trials and hospital authorities in this period than has been recorded to date, and provides a critical and complicated assessment of one of Spain's leading physicians of the nineteenth century, Francisco Javier Balmis (1753–1819).

In July 1790 Nicolás Viana, an itinerant healer from Pátzcuaro in the state of Michoacán, appeared before Archbishop Alonso Núñez Haro y Peralta, director of the Hospital General de San Andrés in Mexico City, claiming to possess a herbal treatment that could heal venereal disease. Viana maintained that over the course of 30 years he had cured thousands of venereal patients suffering from all forms and stages of the disease using a recipe based on two main ingredients—begonia and maguey.^1^ His method, he promised, was faster, cheaper, and less intrusive than the most commonly practised mercurial treatments.^2^ By this stage, a small and apparently successful trial of the cure had already taken place in the Hospital of San Juan de Dios in Mexico City.^3^ Reports indicate that his claims led to a sense of expectation among the medical and clerical authorities in the hospital at the possibility of a home-grown replacement for the usual mercury-based therapies. A claim to possess a new anti-venereal treatment was not, of course, a new phenomenon. That same year an Englishman, Francisco Wates or ‘Francisco El Inglés’, purporting to be a specialist in treating venereal disease, introduced a cure that included sarsaparilla, warm milk and an emetic of tobacco water.^4^ Seven years before Viana's arrival, a virulent polemic over a lizard-based cure for venereal disease had also taken place in the city. The anxiety over the language used in the reports on the lizard cures formed part of a broader context of a new type of reader and consumer of the sciences who increasingly turned to knowledge and scientific practice as a means of defining their role in public life.^5^

The historical, ‘official’ account of the ensuing trials into Viana's remedy, also known as the ‘Beato method’, in the General Hospital of San Andrés and the hospitals of San Juan de Dios, General, and Pasión in Madrid, has long been based exclusively on the published work by the Valencian surgeon, Francisco Javier Balmis (1753–1819), and to some degree on the minutes from meetings held in San Andrés, and the later reports written by the Protomédico, José Ignacio García Jove.^6^ These works have created a picture of a medical community open to testing hypotheses and working to consolidate knowledge; a group largely united despite variance in opinion.^7^

According to these accounts the San Andrés trials comprised a variety of stages, each with an increasingly larger cohort of patients. The initial stage involved 20 male patients and 12 female and lasted for one month, during which time they were treated according to Viana's instructions, without the intervention of other medical staff, and to the exclusion of all other medicines. In the second stage, following a meeting of hospital staff and medical authorities wherein the first results were judged inconclusive, the number tested rose to 40 men and 20 women for over a period of two months. Whilst there are occasional references in the notes that suggest a failure to reach unanimous opinion in absolute support of the cure, the hospital authorities concluded that the ‘Beato method’ was in fact a ‘specific’ for the treatment of venereal disease, and administered it in place of mercury without further delay. It is clear, however, that a majority of patients were once again opting for the mercurial treatments, which caused the authorities to prohibit the further use of any form of mercury in order to promote their chosen method.^8^ Haro y Peralta, convinced of the effectiveness of the cure, commissioned Balmis to bring samples of the Maguey and Begonia plants to Madrid to undertake further trials to persuade the Spanish government of the usefulness of the American discovery. Balmis dutifully travelled to Madrid in 1792 where he undertook a number of trials that led to his publication of the previously mentioned *Demostración de las eficaces virtudes* (1794).

The current study seeks to highlight the problems inherent in privileging successes above failures in our understanding of the past. In other words, to recognise the validity of the path of ‘bad medicine’ tackled by David Wootton, who has argued that:

We know how to write about histories of discovery and progress, but not how to write histories of stasis, of delay, of digression. We know how to write about the delight of discovery, but not about attachment to the old and resistance to the new. We know how to write about successful treatments and lives saved, but not about worthless therapies and lives lost. … Because we only know how to tell one half of the story, the story we could tell is so obviously unsatisfactory that … we usually choose not to tell it.^9^

An increasing number of critical studies into Spanish American medicine and science in the early modern period are moving beyond the need to merely highlight the continent's contribution to world knowledge, and instead dig deeper into the complexities of reception, rejection, promotion, development, and exchange that necessitate recognition of the negative with the positive, and consider errors and failure as well as successes.^10^ With this in mind, I will argue for the importance of including in the historical narrative a long neglected report into the trials written by a contemporary Irish physician-surgeon, Daniel O'Sullivan (1760–*c*.1797), the *Relación histórico-crítica de un supuesto Nuevo Methodo Antivenereo bulgarmente, llamado del Beato*, hereafter referred to as the *Relación.*^11^ This document paints a contrasting picture of a hospital system wherein the exercise of power and authority led to corruption, the misrepresentation of medical data and, in some cases, death, and illustrates the strategies adopted in a struggle to succeed within particular structures of authority and patronage.

I have chosen to refer to this document as an ‘unofficial’ report for two main reasons. First, unlike the sources that have formed our understanding of these events to date, it did not receive recognition from or endorsement by any of the Mexican authorities; furthermore, there is no evidence to indicate that they had any knowledge of its existence. Secondly, despite the fact that it was commissioned by the Royal Academy of Medicine in Madrid, it has remained almost completely untouched and unstudied by scholars since the end of the eighteenth century.^12^

For the purposes of this article I shall focus on the interplay between O'Sullivan, the creation of his *Relación*, and the concept of the international republic of letters of the late eighteenth century as a potential mediator for medical disputes. By setting the *Relación* within this context I hope to highlight the importance of understanding the literary aspects of such medico-scientific texts and explore particularly the means by which authors, such as O'Sullivan, persuade and even manipulate their audience, both real and imagined. This study will engage with O'Sullivan's attempt to promote himself as a reliable narrator by appealing to core ideals within the republic, thus establishing a platform for agreement with the reader. It focuses on two narrative strategies in particular: the employment of contrasting narrative voices within the text, and the creation of enlightened medical heroes with their constituent anti-heroes, such as his depiction of Francisco Javier Balmis.^13^ Key to this approach is O'Sullivan's interest in, and pursuit of, a recognised place in the republic of letters with its constituent value system.

In its ideal form, the republic of letters, was a ‘realm of talent and of thought’; an international, metaphysical, and intellectual community built upon the ideal of the promotion and exchange of knowledge across geographical, religious, and social boundaries.^14^ In many senses it was a forerunner of today's virtual communities whereby individuals sought to increase and share knowledge as well as attain recognition whilst separated by sometimes vast geographical distances. It can be understood as a potentially non-hierarchical space characterised by a particular ‘attitude’, where the ‘practices of intellectual sociability and discourse … were grounded in cultural and epistemological assumptions shared by those who considered themselves to be citizens of that republic’.^15^ These shared assumptions included the importance of links formed by ‘mutual assistance’ and obligation, above and beyond the work of institutions.^16^ Goodman has also argued that in addition to the idea of a ‘pseudo-*Gemeinschaft*’ of the ‘scientific community’, the project of the Enlightenment had given its citizens ‘a greater sense of identity, a greater sense of their own autonomy and high status within their society’ built upon ideals of reciprocity, intellectual cooperation, cosmopolitanism and fidelity to the truth.^17^ As Daston indicated, at the heart of this republic lay the ideal of:

an elite confraternity distinguished by merit in literature, scholarship, and science; by near total freedom of expression …; by equality among members, in defiance of rank and birth; and by tolerance—tolerance that was emphatically religious and incidentally national.^18^

Such sentiments were, in reality, often at odds with the fact that these same individuals were also competing for intellectual accolades and peer approval, with the institutes and academies playing a key role in judging merits and all the ensuing problems these processes entailed.^19^ This world, as Grafton has remarked, formed a ‘palimpsest of people, books, and objects in motion’,^20^ offering potential for a migrant physician in search of career advances in the late eighteenth century.

## O'Sullivan and the Republic of Letters

Born in County Cork, Ireland, O'Sullivan followed in the steps of many young Catholic Irishmen before him, studying Mathematics, Philosophy and Theology at the University of Toulouse, France, in the 1770s.^21^ In 1780 and following his tonsure, he matriculated in Medicine at Toulouse, and, much in the manner of medical students of the time, spent the following five to six years travelling to study at various schools and hospitals in Paris, Montpellier, London, Glasgow, Edinburgh, and Dublin.^22^ In 1786, ‘inspired by tales of Spanish medical excellence’, he travelled to the Iberian Peninsula to further his studies in institutions there, particularly Madrid and Cadiz.^23^ Although it is clear that his applications for positions once in Spain followed a strategy similar to that of those Irish emigrants described by Óscar Recio Morales as ‘active, self-defining individuals’, it is my contention that O'Sullivan's intellectual identity was tightly bound to his French education and perception of its superiority, as well as active expectation of participation in the international republic of letters.^24^ His sense of Irish identity does not find expression in written form at this early point beyond his appeal, when seeking favour, to the long shared history between Irish Catholics and the Spanish, with particular emphasis on his family name.^25^ Nonetheless, once in Mexico he seems to have utilised the Irish connection on two practical counts: on the one hand when seeking witnesses to vouch for his identity and the veracity of his baptismal documents; and on the other, to facilitate sending his reports to Spain.^26^ Within this latter context two links are of particular interest: his correspondence with the leading Irish born physician working in Spain, Timoteo O'Scanlan (1726–96), through whom he maintained links with the Royal Academy of Medicine in Madrid; and his use of the transatlantic networks run by the family of a successful Irish merchant, Patricio Joyes and Co.^27^

The *Relación* was not, however, O'Sullivan's first attempt to rally the interest of the literary republic. In 1789 he had sought limited literary success with an application to publish a literary journal, the *Censor Literario ò Revisor Critico* (*The Literary Censor or Critical Reviewer*). In a manner typical of the publications of the period, he promised a periodical that would highlight all that was beneficial in literature, commerce, agriculture, science, and arts. His application for exclusive rights to the periodical and all income thence deriving received a mixed response from the Spanish authorities. Whilst he was offered muted support to facilitate this project, he was not granted the full economic benefits in the initial stages. The response to his application noted three main concerns: doubts as to the fluency and purity of his Spanish; lack of German language ability, indicating that he could not offer the public any insights into that increasingly important language; and lastly, it was feared that the works printed in the languages in which he was proficient (English and French) would contain errors or ideas relating to doctrine that were not admissible on Spanish soil, necessitating constant vigilance of the content of the proposed publication.^28^ It is not clear whether O'Sullivan ever received word that he had gained consent to publish, as the statement of limited and censored approval was granted only days after he had already set sail for Veracruz with the Spanish army.

Once in Mexico, the language in O'Sullivan's applications for various posts, just as in his *Relación*, suggests a general flexible sense of identity built upon the ideals of the pursuit of knowledge based on reasoned, impartial and critical thinking. This polymorphous approach to identity claims within the republic of letters reflects the migrant path to survival and indicates the particular importance of appealing to those powers and institutions perceived to best further an individual's career, whichever the nation. For an individual in O'Sullivan's situation, the recognition afforded by corresponding membership to a distant and reputable institution such as the Academy of Medicine held potential in a competitive local environment, and for his future should he return to the Peninsula as he seemed to have planned.^29^

Over the course of the seven to eight years he lived in Mexico, he consciously cultivated connections with various centres of knowledge including the Botanical Gardens and the Royal Pontifical University. For the most part, however, his later applications for recognised positions were unsuccessful.^30^ Although O'Sullivan claims that his lack of success in career progression within Mexico was due to the opposition he had voiced to the Beato method trials, it is also possible that he had fallen victim to ongoing antagonism among various institutions and suffered simply from being a ‘foreigner’.^31^ In the case of the treatment offered to venereal patients in the Hospital of San Andrés, or more precisely, the lack thereof, it is clear that by 1792 he had decided to appeal beyond the local authorities in the person of the Viceroy and turn to his trans-Atlantic connections in the hope that the case would be brought to the highest court in the empire, that of Charles IV of Spain. The means by which he worked to persuade his reader is the subject to which we now turn.

## Promoting Reliability: The Narrator and the Reader

The *Relación* that forms the focal point of this article was one of three reports that O'Sullivan submitted at the behest of the Royal Academy of Medicine.^32^ In the concluding section of the last of these, the ‘Carta cirular’ (Circular letter), directed to his friend O'Scanlan in 1793, O'Sullivan states that he had undertaken all that behoved an *hombre de bien* (honest man) and Christian philosopher using all possible means compatible with a prudent approach, in order to confront the ‘torrent of ignorance’ and the ‘stumbling blocks to progress’ in matters that dealt with the life and health of the citizens.^33^ Despite his intention to write a report on the initial trials in 1791 he explains the delay of nearly two years as he bemoans:

But my friend, time, which consumes everything, had weakened this good intention, setting obstacles in the path to completion; the duties inseparable to this sorry profession which we have both embraced; the little joy that can be gained from working in a country where ignorance, charlatanry, inferiority and deceit play such a key role against honour, talent, education, and truth; the desire to distance myself entirely from an issue that has brought me little more than open persecution, … have made me postpone and nearly forget my original aim to deal with this matter.^34^

The request from the Royal Academy in April 1792 seems to have woken him once more from the ‘state of indifference’ into which he had fallen, turning again with enthusiasm to the task at hand.^35^ O'Sullivan can now sense that the doors to the international knowledge network have opened to him, bringing him a step closer to institutional recognition and the possibilities of reciprocity therein, claiming that:

Yes Sir, knowing the current advanced state of Medicine in Madrid, having received news of so many individuals in whom exact philosophy and healthy criticism is evident, knowing at last that there is a Royal Academy of medicine facilitating gatherings of men who are learned, expert, and trained in observation, who, on the basis of their reflections, have reached the zenith of philosophy, which is the art of doubting, under the scrutiny of such highly qualified men, I did not fear the success of such a cure whose temporary effects are only capable of deceiving the ignorant or incautious.^36^

As is evident, O'Sullivan creates a clear divide between his experiences and perceptions of problematic medical practice in Mexico and what he claims to be the greener fields of the court of Madrid. His language appeals to the enlightened institution from the perspective of one who shares their goals and aspirations on a scientific and philosophical level but is trapped in a world that is not of his making.

This appeal to commonality of interest links us directly to the idea of the reliable author. In their study of narrative structure, Herman and Vervaeck outline the fundamental fact that the success of ‘the reliability and the quality of reading depend on the similarity between the implied author's ideology and the ideology of the reader’.^37^ In the most direct sense then, O'Sullivan's readership comprised the élite that formed the Royal Academy of Medicine who commissioned the report. The final success of his claim within the institution is evidenced by the fact that he was offered corresponding membership in October 1793 on the basis of his work.^38^ We find further direct allusion to this group of ideal readers in his concluding appeal in the *Relación*:

The reports to the Royal Academy of Medicine and the Madrid Royal Protomedicato will put an end to these disorders that have lasted for too long. If, distinguished professors, you who have dedicated your studies and countless efforts to the alleviation and conservation of the public, you who know no other language than the truth, … in conclusion, you, whose consummate science, practice, and insight, prevent you from being deceived by illusions; you, worthy successors of the family of Asclepius, will have compassion on the afflictions of this unhappy public and lay their pitiful groans at the compassionate feet of the throne; you, wise companions who do honour to the medical profession, will pour out the consoling balm of health on the open wounds anxious to receive it, and add to the many glorious titles by which you are distinguished in society, that of Protectors and Consolers of the helpless poor.^39^

The tone of the address is, of course, laudatory as he is appealing to their higher senses for the acceptance and promotion of his account. His words hint at the desire for the content of the report to reach the highest echelons of power through appeal to the king and, therefore, a wider circulation outside of the Academy and among the educated elite. The structure of his text must, then, seek to match the ideology of the implied reader in terms of enlightened ideals that honour the medical profession. That said, he also gives his reader a reason to continue through the introduction of various narrative hooks. The methods he employs to achieve this latter goal range from the accessible format of the report—77 folios, divided into 26 reasonably short, numbered sections and a final conclusion—to the choice of content for each section.^40^ It moves from a factual assessment of the history and structure of the hospital with an outline of the history of venereal disease and its many treatments, to an assessment of literature charting the reoccurring practice of charlatanry, and a detailed account of the various stages of the Beato method trials. Within the chronological pattern, the events of the trials are driven forward by the insertion of a critico-satirical analysis of the main characters, their motivations and actions. As the plot thickens in terms of the levels of deceit and manipulation, O'Sullivan introduces little ‘cliff hangers’ at the end of each section, drawing the reader into the story and promising a denouement that never truly materialises as the outcome of the action is still ongoing at the time of writing.

From the outset, he prepares his reader to accept certain premises in the unfolding account through the inclusion of three specific quotations on the title page taken from Virgil, Cicero and Horace. His appeal to the authority of classical texts draws the reader's attention to three central factors relating to the narrator: first, a forewarning of the disturbing content of the narrative that lay ahead and his key involvement in these events, ‘Quaeque Ypse miserrima vidi … *et quorum pars magna fui*’;^41^ second, the resulting sense of responsibility to account for what had happened, ‘Quis nescit primam hanc esse Historiae legem, ne quid falsi dicere audeat; deinceipsne quid veri non audeat’;^42^ and finally, the qualities of character of the narrative's ‘hero’ and *homme ilustré* in the face of all opposition:

Yustum et tenacem propositi Virum / Non Civirem Ardor prava jubentium, / Non Vultus instantis, Tiranni / mente quatit solida / Si fractus illabatur orbis, / Ympavidum ferient Ruinae.^43^

The reader is thereby made aware that terrible events have taken place and that the account that follows is linked to a source that embodies all that is truthful; a narrator certain in his perceptions as a result of experience that has come at great personal cost. The underlying suggestion is, therefore, that the reader may trust the narrator who indicates his critical awareness of these judgements and bases his account not only on the act of ‘seeing’ as ‘eyewitness’ but also ‘perceiving’ through the use of reason.

The *Relación* is, in fact, peppered with the juxtaposition of images of light and dark, sight and blindness, reality versus pretence, all resonant of reason and unreason respectively as he plays to the enlightened concepts of serving the public good.^44^ The emphasis on true sight and the practice of reason or informed sceptical analysis builds upon an *autoptic* rhetorical structure wherein the report becomes an autopsy of medical practices and a veritable dissection of the private interests of those involved in the trials.^45^ The overlap between medical and historical terminology is highlighted by Catherine Darbo-Peschanski's explanation that:

autopsy or personal visual experience, which is implicit in the very etymology of the word *historiē*/*a*, [is] directly derived from the noun *histōr*, itself derived from the root *wid meaning ‘see’ and which also gives the verb *oida*, ‘I know.’ Just as *histōr* means ‘the one who knows because he has seen’…, so *historiē* would be, or would prepare one for, a knowledge founded more specifically on visual observation …^46^

The role of the witness in laying bare the truth in classical historical accounts is similarly echoed in Guido Schepens's study of the Roman historian Ammianus, whose approach, he argues, was a, ‘… “classic” formulation of the method of personal inquiry in history: it envisages *veritas* as the result of a process of research and evaluation (*scrutari*) through autopsy or the careful interrogation of participants in the events'.^47^

For O'Sullivan, the play of visual language and references to classical texts alludes to a continuation of the tradition of Renaissance emphasis on firsthand reporting in historiographical writing. Both Pagden and Greenblatt, building on de Certeau's commentary to Montaigne's ‘Of Cannibals’, have noted the use of the *autoptic* imagination as a key element in early modern accounts of the New World.^48^ Pagden asserts that the use of first person utterances, ‘I saw’, ‘I heard’, etc., are ‘an appeal to the authority of the eye witness, to the privileged understanding which those present at an event have over all those who have only read or been told about it’.^49^ If we follow Greenblatt's argument that ‘the eyewitness possesses the truth and can simply present it: he who has not seen for himself must persuade’, then O'Sullivan's presence at the trials and his personal interaction with the main characters is in itself a strong foundation on which to base his reliability in the mind of the early modern reader.^50^ Furthermore, O'Sullivan adds the emerging late eighteenth-century rhetoric of the trained ‘philosophical’ traveller or observer promoted by such as Raynal's *Histoire philosophique et politique* (1770) and Adam Smith's *The Wealth of Nations* (1776).^51^

The idea of anatomical scrutiny, or the detailed interrogation of all the constituent parts, is a central theme on which he bases his approach:

In history, the facts are like the bones of the skeleton; the chronological order and the natural transition from one subject to another form the joints and the ligaments that bind them; the accidents, small circumstances, with their effects and consequences, are the vessels, membranes, muscles, and other elements that form the natural contour and make History's body beautiful; the truth forms its character, and critical ability provides the senses. But this body with all these requisite parts is still no more than a cadaver, to bring it to life we need to express the particular interests, and the secret passions of the actors, these are the nerves, this the vital principle that sets the whole machine in movement. This is the most difficult aspect of the role of the Historian, and more so if he is dealing with contemporary events.^52^

The difficulties of dealing with live and sensitive issues (the passions and interests) come clearly to the fore in his allusion to the nervous system alongside a recognition that none of the other elements make sense without an attempt to understand these factors. Should the reader have forgotten the numerous struggles the narrator has experienced, we are brought back to consider his initial warnings from the title page and the responsibility that results from such knowledge. O'Sullivan's narrative techniques therefore consciously frame the information he provides and turns the reader's gaze in a particular direction, one of which was the creation of the ‘hero’ and the ‘anti-hero’.

## The Creation of the Medical Hero and Anti-hero

It is important to recognise texts such as O'Sullivan's as examples of literature in the broader sense, especially when taken within the context of his literary ambitions in the republic of letters. As part of the structural strategies employed to engage the reader, O'Sullivan creates a narrative that moves between the extradiegetic and homodiegetic. That is, on some occasions the narrator stands above the events he narrates (extradeigetic), but as part of the *autoptic* imagination he must show that he has also experienced them and is a player in them (homodiegetic). Such an approach means that although at times the character of ‘Dr O'Sullivan’ in the third person is not visible, he is continually present in the text as the omniscient narrator. The homodiegetic narrator, as an involved character, situates himself in a central role in the action (autodiegetic), whilst the extradiegetic narrator is a distanced, impartial observer (allodiegetic).^53^ This method facilitates O'Sullivan's conception of the role of the historian and the reliable, detached, and disinterested voice that stands outside of the action, providing the balanced, critical assessment whilst also promoting the immediacy and reliability of the eye-witness, allowing him to underline his personal efforts in the struggle for the public good. We find this exemplified in the confrontation between O'Sullivan and the proponents of the Beato Method when the cure is first discussed among the hospital staff:

The Beato's claims and the certificates of authenticity he provided in no way persuaded Dr O'Sullivan … he added that no charlatan had ever presented himself before the public without a thousand statements supporting the benefits of his secrets. … These are the reasons, said Dr O'Sullivan, ‘that led me to withhold judgement in this matter, and do not permit me to continue with you, esteemed gentlemen, in the hopes I see you have formed; only experience can overturn my decision and no one would be happier than I should it be proven, for no one could be more completely convinced of the value of a method that at so little cost could destroy *cito tuto et jucunde*^54^ this scourge of humanity; I would go as far as to say that if as few as half the claims made by the Beato were confirmed, the cure would, for me, be a most valuable invention, and I do not doubt that the Public would gain much good from it. But however much I wish it to be true I am even more aware of the deceit should it be false, and as a result I am ever more aware of the need to stay undecided until experience makes the decision clear.’^55^

Both the extradiegetic narrator and the homodiegetic narrator are present. The use of indirect speech when preparing the broader setting of the confrontation and direct speech with the inclusion of quotation marks serves to bring the reader's focus to key arguments. This strategy emphasises his role as the detached, disinterested observer withholding personal opinion and following the route of trial and experience as the basis of scientific practice. The opposite is then inferred of his opponents. The criticism is, of course, that they have already reached their conclusions about the veracity of the cure without waiting for the outcomes of the trial—the lack of proper scepticism. He thus stands out from his opponents and his words are given greater bearing through the repetition of first person utterances. The narrator's use of direct and indirect speech as a means of consciousness representation should lead the reader to question the ‘relationship between representing agent and the one who is being represented’.^56^ The introduction of quoted monologue in direct form reveals a character's thoughts and allows the narrator to ‘cover up his presence’ but also opens up the possibility that he is manipulating the reader who cannot necessarily verify whether or not the words were spoken in exactly such a way or with such clear intent as portrayed in the text.^57^

O'Sullivan often follows a two-pronged approach to questioning the reliability of his opponents. The first step is to outline moral or character weakness and then to build on this to question their ability to reason and work for the common good, and a lack of objectivity. This is evident from the opening section of the *Relación*, in which he lists the physicians, surgeons and administrative staff in each area of the hospital. Included in the lists of staff in the Venereal wards we find ‘Dr Don Daniel O'Sullivan of the Universities of Toulouse, Montpellier, and Edinburgh’ (highlighting his European education and links), followed by a note, wherein the reader is told that:

The former Chief Surgeon of this Department [Venereal Disease], Don Francisco Balmis, had been forced by the Lord Viceroy to obey the repeated Royal Orders issued as a result of his wife's pleas that he return to Spain. … the Archbishop proposed the provisional appointment, of Doctor O'Sullivan to the post, who had been highly recommended and of whom His Excellency had formed a most favourable opinion. Although for various reasons this newly arrived physician did not consider it to be noble employment, he gladly accepted the post as much in recognition of the Lord Archbishop's good intentions as out of an intention to dispel the belief that the treatment for Venereal disease and the use of Mercury were the purview of surgeons, and that the physicians did not understand such matters …^58^

The characterisation of Balmis in this extract and in what follows stands in clear contrast to the portrayal available in the majority of scholarship on this prominent Valencian surgeon, and later physician. Known largely for his role in the Royal Philanthropic Vaccine Expedition of the nineteenth century, sponsored by Charles IV, the focus on the importance and successes of this venture has often created a view of Balmis that overlooks the shadier aspects to his career trajectory.^59^ Fêted in the epic poetry of his time and celebrated still for his role in the fight against smallpox, this later success has led many to read the *Demostraciones eficaces* (1794) in the belief that he was present during the Mexican trials and a key player in the discovery of the cure.^60^ Little, if any, mention is ever made of his role in promoting a cure that was eventually discredited.^61^ If we return to Wootton's critique that we ‘know how to write about successful treatments and lives saved, but not about worthless therapies and lives lost’, then the insights provided by O'Sullivan into this key figure of the Spanish nineteenth century should surely go some way toward addressing such lacunae.^62^

The image of the desperate Spanish surgeon sent scuttling back to Spain to appease the unremitting demands of his wife, allows O'Sullivan to present himself by contrast as a highly educated, purpose-driven individual, who accepts the position offered for higher reasons than his own personal gain. Whilst this could be read as the hyperbole of a university-educated physician over and against a surgical practitioner, his wife had in fact written to the king appealing for the return of her husband due to the total lack of financial support or communication she had received from him since February 1789.^63^ José Tuells has also highlighted the problematic relationship between Balmis and his wife that substantiates O'Sullivan's portrayal.^64^ Tuells also indicates that Balmis had a penchant for actresses, in particular one Antonia San Martín, who it would seem was treated by O'Sullivan for venereal disease, and warned by him to stay clear of charlatan cures.^65^ The questionable aspects of Balmis's character are further undermined by the suggestion that he had directly attempted to discredit O'Sullivan's abilities to treat venereal disease in order to place in post an individual who had agreed to pay him, Balmis, a percentage of his salary whilst *in absentia.*^66^ O'Sullivan thus singles him out as disreputable, lacking in trustworthiness, seeking financial gain at the cost of the medical community, and of questionable moral standards.

This early attempt to discredit the Spaniard is important as it sets the scene for O'Sullivan's later vitriolic criticism that centred on Balmis's unexpected and complete reversal of opinion, moving from outright vocal opponent of the Beato method to primary supporter and eventual ambassador of the cure, a move, we are told, that was driven by Balmis's desire to protect his career interests. O'Sullivan claims that Balmis had been under the continuous scrutiny of both the Viceroy and the Protomedicato since his arrival in Mexico, that he had failed to provide proof of the authenticity of his medical training and avoided tribunals to which he had been summoned. When proof of his training was finally produced, these merely licensed Balmis as a romance surgeon limiting his practice to such an extent that he could not even let blood until he had gained further accreditation.^67^ In light of these facts the Viceroy gave Balmis an official warning against making further false assertions. On his return to Mexico in 1791, without the protection of his patrons in the Gálvez family and with little to no credibility within the medical community or among the public, the Spaniard was desperate to find a means to secure his career.^68^ For the purposes of O'Sullivan's narrative, the argument is clearly that Balmis had falsely inflated his standing in the medical and surgical communities. O'Sullivan, moreover, argues that not only had Balmis never ‘warmed the seats of the universities of Paris and Montpellier for even half an hour’, as he had claimed, but his entire knowledge of that country was based solely on his friendship with a French hairdresser.^69^ The narrator thus lays bare layer upon layer of deception, persistently attempting to undermine any claims to credibility by Balmis, both in his private life and in the public sphere.

There can be little doubt that through these techniques he was aiming to raise questions regarding the suitability of Balmis, setting him on the side of unreason and even deception and, by contrast, promoting his own position as potential arbitrator for the truth. At this time Balmis was promoting the Beato method in Madrid possibly under the protection of the powerful figure of Manuel de Godoy (1767–1851), Duke of Alcudia, royal favourite and first secretary of state in 1792. O'Sullivan's criticism may have been aimed at discrediting Balmis in Spanish medical circles, however, I would suggest that the word of an Irish physician in distant Mexico may have proven ineffective in light of Balmis's powerful patron. Further documentation is necessary to ascertain the validity of many of O'Sullivan's arguments, yet he offers a fascinating insight into the complexity and the drive for ambition of these early medical practitioners and the need to understand the role of the patronage system within which they worked.

Balmis was not, of course, the only powerful figure to receive O'Sullivan's ire. As his account moves from the initial introduction of the character of Viana as a *curandero*, through to processes followed by his supporters in the hospital, we find continuous analogies between dramatic performance and puppetry as a symbols of deceit and manipulation.^70^ In his description of the early meeting to assess the extent to which patients had been cured, O'Sullivan records the following:

All the patients entered singing and dancing, pouring blessings over the Lord Archbishop and Don Nicolas (for the Beato had now reached such levels of respect as to deserve this form of address), proclaiming in loud voices that they were cured and well. This farce was much applauded by the Beato's supporters but any philanthropist taking the opportunity to peek behind that curtain to see the means by which they moved the puppets in this pantomime could not help but be filled with indignation … each one had been given a role to play in this farce. The performance could not have been better designed to convince the Lord Archbishop, body and soul, in defence of this cause, and to instil fear in those practitioners who may have spoken out against the cure and whose silence is now assured.^71^

Further allusions to spectacle and disguise become evident in the discussions that follow amongst the hospital staff:

The role played by Dr Jove in this meeting was very strange. Throughout the entire discussion he remained completely silent, as if he were adopting a stance of complete neutrality. Whenever his opponents directed their arguments to him, he pretended not to hear and left the task of responding to the Archbishop, only encouraging him now and then with a little comment or other, or with some sign of approval to show that he had referenced some term of medical authority well. … No doubt they had adopted a strategy of putting Dr Jove's words in the Archbishop's mouth in the hope of giving them more authority and opening them up to fewer responses; but whatever their intention, it could only have been laughable to see a Master and Doctor, a University Professor and Protomédico remain silent in a meeting with medical staff that dealt with trials in which he himself had been named observer, and an exalted Prince of the church debating these questions with the same zeal and ardour he might have used to defend the consubstantiation of the Divine Word in an argument with an Arian.^72^

There is little doubt that García Jove, President of the Protomedicato, features heavily as one of the main ‘anti-heroes’ and a subtle manipulator on many occasions in this account.^73^ O'Sullivan's attitude to the Archbishop, however, is much more ambiguous. The descriptions vary from direct attacks on his tactics of bullying and intimidation of hospital staff to excusing him on the basis of naiveté and a blindness caused by his desire to promote the public good. Conscious, perhaps, that a critical appraisal of an Archbishop would not sit quite so well with his readers in the Spanish Academy as that of a mere physician, he is quick to temper his language in his conclusion and explain Haro y Peralta's angry outbursts as resulting from an overdeveloped sense of concern for the patients and his hospital. The Protomédicos, however, receive no such recommendation. They are described variously in terms of physical, intellectual, and moral grotesque highlighting directly their perceived limited or lack of ability to practise enlightened medicine, especially within the context of the trials,

Dr Giral, President of the Protomedicato, an octogenarian in whom it is not surprising to note the same weakness of spirit as of body; Dr Rada, docile by nature, lacking in decision, one of those men who prefer to be led rather than be concerned with forming their own opinions, a characteristic that is common in the people of this land; and Dr Jove, a subtle and active personality who, as Protomédico de Merced, is recognised as the mind and soul that directs and encourages all the activities of that triumvirate.^74^

The lack of proper care by the Tribunal is emphasised by the further references to García Jove asserting that he was consistently working to fulfil his own particular ends by whatever strategy necessary, largely absenting himself from the ward rounds in the hospital. Giral is described as quite literally blind and too heavy to be able to stand for any length of time, and as such incapable of performing the ward rounds required of him in the trials.^75^ The metaphorical notion of blindness within this comment is also quite clear. Rada, in the meantime, who was acting on behalf of all three, examines the patients declaring that ‘some still had a wee fever, another a wee cold, this one a wee nothing, and that one some other wee symptom’.^76^ These expressions depict either a lack of knowledge or proper care in assessing the true condition of the patients and underline the more satirical elements in O'Sullivan's criticism of the lack of acceptable medical procedures in place. We should also note among the scathing descriptions of the Protomédicos, a singular example of O'Sullivan's opinion of the people of New Spain as weak-willed, or lacking in decisiveness, and easily led, that seems to tie directly to generalised ideas of degeneracy and the inferiority of the Americas.^77^ This attitude tallies with his overall approach that sides more on his perception of European superiority in education and character over that of his New World contemporaries. A strong explanation, we could conclude, as to why he failed so significantly to adapt himself into the local structures.

In sharp contrast to these morally and professionally questionable supporters of the Beato method, we find the few who openly opposed the decisions taken during the trials; most notably, Alejo Ramón Sánchez.^78^ O'Sullivan depicts Sánchez very much as a martyr in the pursuit of medical truth as well as an *homme ilustré.* Victim of yet one more act of deceit on the part of the Beatistas, Sánchez is blamed for the death of a soldier who had presented with severe symptoms, particularly tumours in the parotid glands, but whose condition had been ignored in the previous meeting to assess the status of the venereal patients. According to O'Sullivan the ineffective cure allowed the tumours to increase resulting in the suffocation of the patient, yet the Beatistas were quick to cast the ‘venom of this catastrophe against the opponents of the method.’^79^ Sánchez, then, became their scapegoat. O'Sullivan's depiction of a man of sense of honour and faith embodies an enlightened ideal both in character and professional commitment: Sánchez was ‘an excellent teacher, a man of talent, intellect, and commitment, an adornment to his profession, the delight of society’, of ‘virtuous conduct’, a ‘constant friend’, ‘excellent husband’ and ‘loving father’ who refused to be cowed and, instead, remained to stand against the false testimony of his aggressors. In the event, he died from a severe case of colic before his case could ever be brought to trial.^80^ Two other colleagues receive similar if briefer praise wherein the moral character of the individual is closely tied to their enlightened approach to medicine: Mariano Aznares is described as working with ‘method and energy’ and Josef Ferrer as a man of ‘no self-interest but of modesty and moderation’.^81^ O'Sullivan places himself alongside Sánchez claiming the medical and moral high ground, and arguing that had he been in a similar position:

he would not have become demoralised, rather solus contra omnes, he would have appealed to the higher court of the republic of letters, and confidently awaited the decision, declaring in the interim, vertute mea me involvo.^82^

Where Sánchez quite literally has become a martyr to the truth, O'Sullivan depicts himself in similar light as a stoic and a martyr in terms of the negative impact on his career and his eventual dismissal from the hospital.^83^

## Conclusions

In the final two sections of the *Relación* O'Sullivan provides one last reflection on the goal of his report. Throughout, he has argued for the need to educate and inform both the public and the hospital staff regarding the implications of the new cure. He promotes this idea in the hope that they will put aside their ‘blindness’ and become aware of the deceit to which they have been victims. It seems that the ‘bitter wailing’ of the people as a result of the treatment had eventually galvanised the council of the city of Mexico into action to set up some form of enquiry.^84^ No results were forthcoming at the time of writing the *Relación*, but in the interim, O'Sullivan clearly expects the Royal Academy of Medicine and the Madrid Protomedicato to exercise some influence on the Mexican situation should it still be necessary by the time they have read his reports.

Further work is necessary to understand the extent of influence and relationships that existed, if any, between the various Tribunals of the Protomedicato in each viceroyalty as well as in Spain. It is possible that O'Sullivan believed that with the support of the Academy, influence could be exercised on a political level between the court in Madrid and the city council or viceroy in New Spain. In this way, he acted in the belief that writers ‘as knowledge and opinion shapers formed a sort of power which was as formidable as that of organised government’.^85^ However, the events that followed indicate that the *Relación* had little impact outside of the walls of the Academy. Setting aside his role as narrator in the concluding section, O'Sullivan writes, that:

It has not been my intention to defend or harm anybody's reputation. … I have related the events exactly as they took place, it has not been possible to omit any without lessening the truth and harming the cause in defence of which I have undertaken this work, that is, the benefit to humanity and the honour of the medical profession …^86^

Building on this claim that he is working to achieve a practical, intellectual and humanitarian goal, O'Sullivan concludes by projeting an ironic vision of outcomes to the trial wherein the improbable becomes the possible and the medical community becomes a *mundo al revés* or world upside down. Should the esteemed colleagues in Spain find the cure to be radical and effective, O'Sullivan proclaims, he will wholeheartedly congratulate the Archbishop when he is given a cardinal's purple robe; he will be overjoyed for Balmis when he becomes surgeon to the king's chamber, or obtains the title of physician; and he will congratulate the Beato when he receives a little pension with exclusive privileges to sell Begonia roots throughout the king's domains.^87^ Little could he have dreamt that in terms of the career trajectory for Balmis at least, the unimaginable became the reality.^88^

O'Sullivan closes his appeal with the obvious expectation that within the republic of letters, and most specifically the Academy of Medicine, the ideology that has informed his criticism will create a bridge to his readers' understanding. The possibility of recognition within this institution no doubt excited his anticipation of the system of reciprocity and mutual benefits, as well as leading to the promotion of the public good. Yet despite his inclusion as a corresponding member of the Academy in 1793 and their agreement with his analysis, it is unclear why little or no action was taken in regard to the continued promotion of the cure. These issues form part of further investigation necessary into the systems of patronage in the practice of medicine in the early modern world. And if, as Daston has argued, recognition, once given within the illustrious network of the republic of letters, did not remain at an individual level, but reflected on the nation, how does this relate to the space occupied by migrant physicians such as O'Sullivan and their survival strategies?^89^

## Footnotes

1 The alcoholic extract of the maguey was used as a sweating agent and the begonia was ground up as a purgative. Several other ingredients were also added, including a third formula comprising sarsaparilla and water, lemon, and copal of Campeche, sassafras and sugar. Other elements initially included were snake flesh and a flower known as ‘rosa de castilla’ (Archivo General de la Nación, México (AGN), Bienes Nacionales, 593, exp. 3). The recipe was later simplified by Francisco Javier Balmis recorded in his account of trials held in Madrid, *Demostración de las eficaces virtudes nuevamente descubiertas en las raíces de dos plantas de Nueva España, especies de agave y de begonia, para la curación del vicio venéreo y escrofuloso, y de otras graves enfermedades que resisten al uso del Mercurio, y demás remedies conocidos* (Madrid, 1794), 10–18. This was then further translated into Italian and published in Rome in 1795.

2 For further study on treatments of venereal disease and charlatanism see for example: Claudia Stein, *Negotiating the French Pox in Early Modern Germany* (Farnham: Ashgate, 2009); Kevin Brown, *The Pox. The Life and Near Death of a Very Social Disease* (Gloucestershire: Sutton Publishing, 2006); David Gentilcore, *Medical Charlatanism in Early Modern Italy* (Oxford: Oxford University Press, 2006); Kevin P. Siena, *Venereal Disease, Hospitals, and the Urban Poor: London's ‘Foul Wards’, 1600–1800* (Rochester, NY: University Rochester Press, 2004); Roy Porter, *Quacks. Fakers and Charlatans in Medicine* (Wiltshire: Tempus, 2003); Claude Quétel, *The History of Syphilis,* Judith Braddock and Brian Pike (trans) (Baltimore: Polity Press, 1990).

3 In 1779, Alonso Núñez Haro y Peralta successfully petitioned to be given responsibility for establishing a hospital in what would become the Hospital General de San Andrés. In 1786 the Viceroy granted him complete control of the running and financing of the hospital. This meant that the hospital was not answerable to the civil authorities for either the administration or financial practices. See Josefina Muriel, *Hospitales de la Nueva España: fundaciones de los siglos XVII y XVIII,* 2 vols (México: UNAM, 1991) *II*, 215–39.

4 Luz María Hernández Saenz, *Learning to Heal. The Medical Profession in Colonial Mexico, 1767–1831* (New York: Peter Lang, 1997), 238. By the time Wates's activities had been reported to the Protomedicato he had disappeared with any advance payments patients had made to him. The Royal Tribunal of the Protomedicato, based in the city of Mexico, was the official board of health for the vice royalty. It comprised three leading physicians, Protomédicos, two of which held the first and second chairs of medicine in the Royal Pontifical University of Mexico, and the third named by the Viceroy. Their main function was to certify medical practitioners and the teaching of medicine and to regulate medical practice and new medical knowledge. See John Tate Lanning, *The Royal Protomedicato: The Regulation of the Medical Profession in the Spanish Empire,* ed., John Jay Tepaske (Durham, NC: Duke University Press, 1985), and the articles in the special edition, ‘The Royal Protomedicato Tribunal in the Hispanic Monarchy, 1593–1808’, *Dynamis*, 1996, *16*.

5 Miruna Achim, ‘Making lizards into drugs: the debates on the medical uses of reptiles in late eighteenth-century Mexico’, *Journal of Spanish Cultural Studies*, 2007, *8*, 169–91, 167 and 183. See also Miruna Achim, *Lagartijas medicinales. Remedios americanos y debates científicos en la Ilustración* (México: CONACULTA, 2008).

6 Francisco Javier Balmis, *Demostración de las eficaces virtudes* (1794); Archivo General de la Nación, México (AGN), Bienes Nacionales, 593, exp. 3.

7 See most recently Alba Dolores Morales Cosme, *El Hospital General de San Andrés: la modernización de la medicina novohispana (1770–1833)* (México: UAM, 2002).

8 Balmis, *Demostración de las eficaces virtudes* (1794), 1–19; AGN, Bienes Nacionales, 593, exp. 3.

9 David Wootton, *Bad Medicine: Doctors Doing Harm Since Hippocrates* (Oxford: Oxford University Press, 2007), 15.

10 Amongst the most recent books published relating to medicine and science in Latin America in the early modern period, such as the aforementioned work by Achim, we could note, Daniela Bleichmar, *Visible Empire. Botanical expeditions and visual culture in the Hispanic Enlightenment* (Chicago: University of Chicago Press, 2012), Adam Warren, *Medicine and Politics in Colonial Peru. Population Growth and the Bourbon Reforms* (Pittsburgh: University of Pittsburgh Press, 2010), Daniela Bleichmar, Paula DeVos, Kristin Huffine and Kevin Sheehan (eds), *Science in the Spanish and Portuguese Empires, 1500–1800* (Stanford: Stanford University Press, 2009).

11 Daniel O'Sullivan, *Relación histórico-crítica de un supuesto nuevo methodo antivenereo bulgarmente, llamado del Beato, introducido en el Hospital de San Andres de Mexico en el Año de 1790, y establecido con exclusion del Mercurio en el de 91. La dirige en el de 92 a la Real y mui ilustre Academia Medica matritense un doctor en medicina residente en dicha ciudad*, (A historico-critical account of a supposed new anti-venereal method commonly known as the Beato method, introduced in the Hospital of San Andrés in Mexico in 1790, and established to the exclusion of mercury in 1791. Addressed to the Royal and illustrious Academy of Medicine in Madrid in 1792 by a medical doctor residing in that city). Real Academia Nacional de Medicina (RANM), Madrid, Serie Documental, Fol. 40, leg. 14c, doc. 67. The use of underlining is presented as in original manuscript, spelling and typography are also as in the original document, translations from the Spanish are my own.

12 Miruna Achim mentions that O'Sullivan's report refers to the lizard remedy from the 1780s, *Lagartijas medicinales*, 196; The only other extended use of details from this manuscript can be found in Nicolás Léon, *Biblioteca botánica-mexicana. Catálogo bibliográfico, biográfico y crítico de autores y escritos referentes a vegetales de México y sus aplicaciones, desde la conquista hasta el presente. Suplemento a la materia médica mexicana publicada por el Instituto Médico Nacional* (México: Oficina Tip. de la Secretaría de Fomento, 1895), 29–54. León uses extracts of the *Relación* as a critique of the scientific value of Balmis's 1794 publication, the *Demostración*.

13 The details discussed in this article comprise a small part of a much more extended analysis in my current project that explores O'Sullivan's role in the anti-venereal trials, the treatment of soldiers, and the pursuit of patronage and self-promotion in the Spanish empire.

14 Anon, *Histoire de la république des lettres en France* (Paris, 1780), quoted in Dorinda Outram, *The Enlightenment* (Cambridge: Cambridge University Press, 1997), 21.

15 Dena Goodman, *The Republic of Letters: A Cultural History of the French Enlightenment* (Ithaca, NY: Cornell University Press, 1996), 3.

16 Anne Goldgar, *Impolite Learning. Conduct and Community in the Republic of Letters, 1680–1750* (New Haven, CT; London: Yale University Press, 1995), 13.

17 Goodman, *The Republic of Letters*, 23.

18 Lorraine Daston, ‘The ideal and reality of the republic of letters in the Enlightenment’, *Science in Context*, 1991, *4*, 367–86, 374.

19 *Ibid.*, 379.

20 Anthony Grafton, ‘A Sketch Map of a Lost Continent: The republic of letters’, *Republics of Letters: A Journal For the Study of Knowledge, Politics, and the Arts*, 2009, *1*, 1–18, 5: <http://rofl.stanford.edu/node/34>, accessed 21 November 2012.

21 Extensive work has been undertaken on the area of Irish migration in relation to Europe and more limitedly in the Iberian Empire. For a particularly broad range of scholarship see the work of Marian Lyons and Thomas O'Connor for the series Irish in Europe, Dublin, Four Courts Press, including, *Irish Communities in Early Modern Europe* (2006) and *Strangers to Citizens: The Irish in Europe, 1600–1800* (Dublin: National Library of Ireland, 2008). With a focus on Irish migration and the Spanish military see, E. García Hernán and Ó. Recio Morales (eds), *Extranjeros en el ejército. Militares irlandeses en la sociedad española 1580–1818* (Madrid: Ministerio de Defensa, 2007) and in the Iberian Atlantic see, Igor Pérez Tostado and Enrique García Hernán (eds), *Irlanda y el Atlántico ibérico: movilidad, participación e intercambio cultural, 1580–1823* (Valencia: Albatros, 2010). For an emphasis on migration and medicine see, James Kelly and Fiona Clark (eds), *Ireland and Medicine in the Seventeenth and Eighteenth Centuries* (Farnham: Ashgate, History of Medicine in Context Series, 2010).

22 For excellent discussion of the movement between centres of medical education in this period see: Ole Peter Grell, Andrew Cunningham and Jon Arrizabalga (eds), *Centres of Medical Excellence? Medical Travel and Education in Europe, 1500–1789* (Farnham: Ashgate, 2010).

23 Archivo Histórico de la Facultad de Medicine, Universidad Autónoma de México (AHFM-UNAM), leg. 2, exp. 6. Whilst not wishing to detract from valuable examples of Spanish medical practice in this period, we should, of course, note that he makes these claims to the excellence of medical training Spain in a document requesting certain privileges within that same Empire, and should therefore also be understood within the context of self-promotion. Archivo General de Simancas (AGS), Guerra Moderna, leg. 6974; AGN, Historia, 3, fol. 33; AGN, Universidad, Libro de Claustros, t. 27, 109r–211v. See also L. W. B. Brockliss and Patrick Ferté, ‘Prosopography of Irish Clerics in the Universities of Paris and Toulouse, 153–1792’, *Archivium Hibernicum,* 2004, *58*, 7–166, 100.

24 Óscar Recio Morales, ‘Irish émigré strategies of survival, adaptation and integration in seventeenth- and eighteenth-century Spain’ in O'Connor and Lyons (eds), *Irish Communities in Early Modern Europe*, 240–66.

25 See, e.g., Claire Caroll, ‘Irish and Spanish cultural and political relations in the work of O'Sullivan Beare’, in Hiram Morgan (ed.), *Political Ideology in Ireland, 1541–1641* (Dublin: Four Courts Press, 1999), 229–53.

26 One of the witnesses called to validate O'Sullivan's petition for permission to practise medicine was Coronel Marcos Keating of the Royal Artillery (AHFM–UNAM, Leg. 2, exp. 6, fols 7r–7v) and at least one of his reports to the Royal Academy of Medicine was sent via Patricio Joyes and Co. (RANM, Leg. 14c, doc. 68). Patricio Joyes (Joyce), from County Galway, Ireland, was the founder of one of the most powerful banking firms in eighteenth-century Spain. See Óscar Recio Morales, *Ireland and the Spanish Empire, 1600–1825* (Dublin: Four Courts Press, 2010), 257; Mónica Bolufer Peruga, ‘Inés Joyes y Blake: una ilustrada, entre privado y público’ in Rosa Capel (ed.), *Mujeres para la historia. Figuras destacadas del primer feminismo* (Madrid: Abada Editores, 2004), 27–55.

27 Surprisingly little has been written about O'Scanlan to date, but for some further details see: Juan Riera, *Cirugía española ilustrada y su comunicación con Europa. (Estudio y documentos de un influjo cultural)* (Valladolid: Secretario de Publicaciones, 1976); Michael White, ‘The role of Irish doctors in eighteenth-century Spanish medicine’, in Declan M. Downey and Julio Crespo MacLennan (eds), *Spanish-Irish Relations Through the Ages* (Dublin: Four Courts Press, 2008); Mónica Amenedo Costa, ‘Timoteo O'Scanlan: vida pública y privada en el Ferrol ilustrado’, *Revista Garoza*, 2010, *10*, 9–22.

28 He claims to speak to the languages of Ireland and Scotland also. Whether this referred to the Gaelic language or broader regions where English was used is unclear. Archivo Histórico Nacional de Madrid (AHNM), Consejos, 5555(83).

29 In June 1793 O'Sullivan applied for leave of absence to return to Spain to see to personal matters. Permission was granted in October 1793 but O'Sullivan does not seem to have returned to Spain as planned before his death (AGN, Indiferente de Guerra, 385A; AGS, Guerra Moderna, leg. 6966, fol. 231).

30 For further discussion of O'Sullivan's largely failed attempts to promote his career in colonial Mexico see: Fiona Clark, ‘Advancing the medical career abroad: Daniel O'Sullivan (1760–*c*.1797)’, in Kelly and Clark (eds), *Ireland and Medicine*, 197–216. For broader biographical details on O'Sullivan's work as military and hospital surgeon see María Luisa Rodríguez-Sala, *Los cirujanos de hospitales de la Nueva España (1700–1833) ¿Miembros de un estamento profesional o una comunidad científica?* (México: UNAM, 2004), 90, 95–6, 99, 105–6.

31 The authorities of the Pontifical University, in particular, refused to consider him for academic posts on the basis of his ‘foreignness’ (AGN, Universidad, vol. 27, Libro de Claustros, fols 209r–211v.). Hernández Saenz provides an overview of the exercise of power dynamics for physicians and surgeons in the Royal Court, particularly the foreigners, see *Learning to Heal*, 21–128. During this period there were tensions at play between the Royal Pontifical University, the Royal Botanical Gardens, and the compulsory teaching of Botany as part of the medical curriculum. O'Sullivan is linked to all of these areas. For broader discussion of the polemic between the institutions see, for example, Patricia Aceves Pastrana, *Química, botánica y farmacia en la Nueva España a finales del siglo XVIII* (México: Universidad Autónoma de México, 1993).

32 The other documents comprise: ‘Representaciones hechas al Exmo Señor Virrey de México sobre los malos efectos del supuesto especifico del Beato’ (1791–3), (RANM, fol. 16, leg, 14c, doc. 69); ‘Carta circular del doctor Don Daniel O'Sullivan a 25 médicos con sus respuestas y un apéndice sobre el específico antivenéreo del Beato’ (1792), (RANM, Leg. 14c, doc. 68).

33 O'Sullivan, ‘Carta circular’, n.p. The term ‘hombre de bien’ also carries significance as an enlightened man.

34 *Ibid.*, n.p.

35 *Ibid.*, n.p. To date, I have found no trace of the April 1792 letter, or a further letter on the matter written by O'Scanlan in June 1792.

36 *Ibid.*, n.p.

37 Luc Herman and Bart Vervaeck, *Handbook of Narrative Analysis* (Lincoln and London: University of Nebraska Press, 2001), 20.

38 In June 1793 Timoteo O'Scanlan submitted the *Relación* to the Academy requesting that O'Sullivan be considered for membership given the zeal and love for the practice of medicine indicated in his reports (RANM, leg. 1, doc. 22). The final decision by the Academy noted that O'Sullivan had concluded that the cure was doubtful and prejudicial, and that he had censured the actions of both Viana and his protectors with ‘appropriate liveliness’ (RANM, Leg. 4, doc. 169); *Libro de acuerdos de la Real Academia Médica de Madrid*, 1791–1813, 67. Notable among the assessors of the report was the well known physician Ignacio María Ruiz de Luzuriaga, who had trained in France and in Scotland, under Cullen, Hunter and Pott, see Juan Riera, *Cirugía española ilustrada y su comunicación con Europa* (Valladolid: Secretariado de Publicaciones, 1976), 186.

39 O'Sullivan, *Relación*, fol. 75.

40 The main script does not belong to O'Sullivan whose easily identifiable writing can be found in occasional annotations, deletions and the inclusion of a section of final thoughts.

41 O'Sullivan, *Relación*, fol. 1, ‘quaeque ipse miserrima vidi, / et quorum pars magna fui.’ Virgil, *The Aeneid*, Book II:5–6 (the sights most piteous that I saw myself and wherein I played no small part) in Virgil, *Eclogues, Georgics, Aeneid I–VI*, H. Rushton Fairclough (trans) (Cambridge, MA; London: Harvard University Press, 1999), 316–17. The quotations used by O'Sullivan differ at times, for this reason I have chosen to repeat the correct Latin quotations in the corrected form in the footnotes.

42 O'Sullivan, *Relacion,* fol.1, ‘Nam quis nescit, primam esse historiae legem, ne quid falsi dicere audeat? Deinde ne quid veri non audeat?’ Cicero, *De Oratore*, Book II:XV, 62 (For who does not know history's first law to be that an author must not dare to tell anything but the truth?) in Cicero, *De Oratore*, *Vol. I, Books I, II,* E. W. Sutton (trans) (Cambridge, MA: Harvard University Press and London: William Heinemann Ltd., 1968), 242–5.

43 O'Sullivan, *Relación*, fol. 1. This extract provided by O'Sullivan is taken from the following longer quotation: ‘Iustum et tenacem propositi virum / non civium ardor prava iubentium, / non vultus instantis tyranni / mente quatit solida neque Auster, / dux inquieti turbidus Hadriae, / nec fulminantis magna manus Iovis:/ si fractus illabatur orbis, / impavidum ferient ruinae.’ Horace, *The Odes*, Book III, 3, 1–8 (The man of integrity who holds fast to his purpose is not shaken from his firm resolve by hot-headed citizens urging him to do wrong, or by the frown of an oppressive despot, or by the South Wind, that unruly lord of the restless Adriatic, or by the mighty hand of thundering Jove) in Horace, *Odes and Epodes*, Niall Rudd (ed. and trans) (Cambridge, MA; London: Harvard University Press, 2004), 146–7. A further interesting area of study lies in the potential import of O'Sullivan's choice of classical texts and the significance of their original contexts in regard to his observations of the eighteenth-century trials. Such a discussion will take place within the larger project from which the current study is taken but is not possible here.

44 The following list serves as a short example: ‘true light’ (f. 62), ‘he was enlightened’ (fol. 62), ‘open their eyes’ (fol. 59), ‘those events he thought he had seen with his eyes’ (fol. 47), ' eye witness' (fol. 60), ‘illusion or reality’ (fol. 47), ‘general blindness’ (fol. 50), ‘secretly’ (fol. 44), ‘dissimulation’ (fol. 44), ‘pretence’ (fol. 44), and ‘traps’ (fol. 44).

45 One of O'Sullivan's central complaints about the clerics and physicians linked to the General Hospital of San Andrés was that in their rush to prove the cure to be specific they did not ‘know how to doubt’ (saver dudar), O'Sullivan, *Relación*, fol. 17.

46 Catherine Darbo-Peschanski, ‘The origin of Greek historiography’, in John Marincola (ed.), *A Companion to Greek and Roman Historiography*, 2 vols (Oxford: Blackwell Publishing, 2007), I, 27–38, 29.

47 Guido Schepens, ‘History and Historia: Inquiry in the Greek Historians’, in Marincola (ed.), *A Companion to Greek and Roman Historiography*, I, 39–55, 39.

48 Michel de Certeau, *Heterologies. Discourse on the Other*, Brian Massumi (trans) (Minneapolis: University of Minnesota Press, 2010), 67–79.

49 Anthony Pagden, *European Encounters with the New World* (New Haven, CT; London: Yale University Press, 1993), 51; Stephen Greenblatt, *Marvellous Possessions. The Wonder of the New World* (Chicago: University of Chicago Press, 1991), 129.

50 Stephen Greenblatt, *Marvellous possessions*, 129; and Pagden, *European encounters*, 78.

51 See Jorge Cañizares-Esguerra, *How to Write the History of the New World. Histories, Epistemologies, and Identities in the Eighteenth-century Atlantic World* (Stanford, CA: Stanford University Press, 2001), 12 and 20.

52 O'Sullivan, *Relación*, fols. 67–8.

53 For a clear and accessible outline of the different approaches to narrative analysis see Herman and Vervaeck, *Handbook of Narrative Analysis*.

54 ‘Cito tuto et jucunde’ (quickly, safely, pleasantly).

55 O'Sullivan, *Relación*, fol. 17.

56 Herman and Vervaeck, *Handbook of Narrative Analysis*, 23.

57 For variants within these narrative strategies see *Ibid.*, 24–30.

58 O'Sullivan, *Relación*, fol. 3.

59 There is a significant body of literature on Balmis and the expeditions, the most recent include: José Tuells and Susana María Ramírez Martín, ‘Francisco Xavier Balmis y las Juntas de Vacuna, un ejemplo pionero para implementar la vacunación’, *Salud pública mexicana*, 2011, *53*, 172–7; Catherine Mark and José G. Rigau-Pérez, ‘The World's First Immunization Campaign: The Spanish Smallpox Vaccine Expedition, 1803–1813’, *Bulletin of the History of Medicine*, 2009, *83*, 63–94.

60 See, e.g., Manuel José Quintana's epic poem, ‘A la expedición española para propagar la vacuna en América bajo la dirección de don Francisco Balmis’ (1806).

61 In 1895 León used O'Sullivan's text to argue that the most prominent critic during the time of the Madrid trials, Bartolomé Piñera y Siles, was in fact correct in his negative assessment of Balmis's work when he published his *Narración histórica de las observaciones ó ensayos prácticos que se han hecho en los Hospitales de San Juan de Dios, General y Pasión de esta corte* (1792). See León, *Biblioteca botánica-mexicana, 50.*

62 Wootton, *Bad Medicine*, 15.

63 AGN, Reales Cédulas, 143, exp. 120.

64 Despite his 30 year marriage to Josefa Mataix, Balmis claimed to be unmarried in 1803 and 1813. It was not until 1816 that he acknowledged his wife without bequeathing her anything in his wills. See José Tuells y José Luis Duro Torrijos, ‘Los cinco testamentos de Francisco Xavier Balmis’, *Gaceta médica de México*, 2012, *148*, 411–18.

65 See Tuells, ‘Escenarios vitales de Francisco Xavier Balmis (1753–1819), Director de la Real Expedición Filantrópica de la Vacuna’, *Real Academia Medica, Comunitat Valenciana*, 2011, *12*, 1–17, 8–9.

66 O'Sullivan, *Relación*, fol. 3.

67 Tuells has recorded that Balmis was examined in 1777 by the surgeons of the King's Bedchamber and given licence to let blood and pull teeth, amongst other practices. In 1778 he is granted the title of surgeon by the Royal Protomedicato before continuing his career in the Spanish army. See Tuells, ‘Escenarios vitales de Francisco Xavier’, 6–7.

68 O'Sullivan, *Relación*, fols. 58–65. This is a reference to links between Balmis and the family of Bernardo de Gálvez, Viceroy of New Spain (1746–86) and his uncle José de Gálvez (1720–87), who acted as Minister of the Indies (1775–87).

69 *Ibid.*, fols. 61–7.

70 The term *curandero* can be used to refer to a folk healer but within the context of O'Sullivan's report he clearly adopts it to mean charlatan.

71 O'Sullivan, *Relación*, fol. 24.

72 O'Sullivan, *Relación*, 25–6.

73 José Ignacio García Jove, President of the Protomedicato, and physician in the medical wards of San Andrés, had a long, wealthy and colourful career. In 1792 he held the Chair of *Vísperas de Medicina* (Pathology) in the Royal Pontifical University *Calendario manual y guía de forasteros en México, para el año de 1792* (Mexico: Zúñiga y Ontiveros, 1792), 104. Hernández Saenz provides several cases where he was accused of extortion and bribery, see *Learning to Heal*, 47, 49–50, 53–4, 157, 197–9, 234 and Tate Lanning, The *Royal Protomedicato*, 253–4, 286–290.

74 *Ibid*., fols. 35–6.

75 Protomédico José Antonio Giral held the Chair of *Prima de Medicina* (Physiology) at the Royal Pontifical University, *Calendario manual y guía de forasteros* (1792), 104; Francisco A. Flores, *Historia de la Medicina en México desde la época de los indios hasta la presente* 2 vols (México: Secretría de Fomento, 1886), *II*, 90.

76 *Ibid*., fol. 37. I have chosen to use the more colloquial expression ‘wee’ in this translation as a means of expressing the Spanish apocopation ‘ito’ or ‘ita’ at the end of the terms a childishness of approach and minimisation of the importance of the proper terms. In 1792 José Francisco Rada held the Chair of Mathematics in the Royal Pontifical University, *Calendario manual y guía de forasteros* (1792), 104.

77 This argument, that was particularly important in the latter part of the eighteenth century, has a long history but one key work is Antonello Gerbi's *The Dispute of the New World: The History of a Polemic, 1750–1900*, Jeremy Moyle (trans), 1st edn 1955, (reissued and revised University of Pittsburgh Press, 2010).

78 Alejo Ramón Sánchez, educated in the College of Surgery in Cadiz, worked in the surgical wards in San Andrés, *Relación,* f. 4. He was one of the key players in the polemic that took place over the introduction of a lizard cure in 1782. See Achim, *Lagartijas medicinales* and ‘Making Lizards into drugs’.

79 O'Sullivan, *Relación*, fol. 34.

80 *Ibid*., fol. 35.

81 *Ibid*., fol. 40. Mariano Aznares, educated in the University of Zaragoza, held a post as physician in the medical department of San Andrés alongside García Jove. José Antonio Ferrer, educated in the College of Surgery in Barcelona worked as second surgeon with Alejo Ramón Sánchez in the Surgical department, *Relación*, fol. 4. See also Rodríguez Sala, *Los cirujanos de hospitales*, 105.

82 O'Sullivan, *Relación*, fol. 21. ‘Virtuti mea me involvo’, Horace, *Odes*, Ill: 29, 55 (I wrap myself in my virtue) in Horace, *Odes and Epodes*, 214–13.

83 O'Sullivan, *Relación*, fol. 60.

84 O'Sullivan, *Relación*, fol. 75.

85 Outram, *The Enlightenment*, 21.

86 O'Sullivan, *Relación*, fol. 75.

87 *Ibid*., 76.

88 Balmis was granted the title of Honorary Surgeon of the King's Bedchamber in 1795. We should note the José de Flores, whose work on lizard cures had caused the polemic in 1782 was also granted the title of Physician of the King's Bedchamber in 1794.

89 Daston, ‘The ideal and reality’, 380.

